# Exploring the genetic diversity and population structure of *Ailanthus altissima* using chloroplast and nuclear microsatellite DNA markers across its native range

**DOI:** 10.3389/fpls.2023.1197137

**Published:** 2023-11-22

**Authors:** Josphat K. Saina, Zhi-Zhong Li, Boniface K. Ngarega, Robert W. Gituru, Jin-Ming Chen, Yi-Ying Liao

**Affiliations:** ^1^ Key Laboratory of Southern Subtropical Plant Diversity, Fairy Lake Botanical Garden, Shenzhen & Chinese Academy of Sciences, Shenzhen, China; ^2^ Wuhan Botanical Garden, Chinese Academy of Sciences, Wuhan, China; ^3^ University of Chinese Academy of Sciences, Beijing, China; ^4^ Sino-African Joint Research Center, Chinese Academy of Sciences, Wuhan, China; ^5^ Center for Integrative Conservation, Xishuangbanna Tropical Botanical Garden, Chinese Academy of Sciences, Menglun, Yunnan, China; ^6^ Department of Botany, Jomo Kenyatta University of Agriculture and Technology, Nairobi, Kenya

**Keywords:** *Ailanthus altissima*, disturbance, genetic diversity, microsatellites, population structure, species distribution modeling

## Abstract

Understanding how anthropogenic disturbances affect the genetics of tree species is crucial; however, how tree populations in the wild can tolerate these activities remains unexplored. Given the ongoing and intensifying anthropogenic disturbances, we conducted a study using *Ailanthus altissima* to gain new insights into the effects of these pressures on genetic variability in undisturbed and disturbed forests. We analyzed the genetic diversity and population structure of *A*. *altissima* using nuclear (EST-SSR) and chloroplast (cpSSR) microsatellite markers. The genetic diversity across the 34 studied populations based on EST-SSRs was found to be moderate to high (*nH*_E_ = 0.547–0.772) with a mean *nH*_E_ of 0.680. Bayesian clustering, principal coordinate analysis (PCoA), and discriminant analysis of principal component (DAPC) consistently divided the populations into three distinct groups based on EST-SSRs. Allelic combinations of 92 different chloroplast size variants from 10 cpSSR loci resulted in a total of 292 chloroplast haplotypes. The mean haplotype diversity was relatively high (*cpH*_E_ = 0.941), and the mean haplotype richness was 2.690, averaged across the 34 populations of *A*. *altissima*. Values of *F*_ST_ in *A*. *altissima* from chloroplast and nuclear markers were 0.509 and 0.126, respectively. Modeling results showed evidence for population range contraction during the Last Glacial Maximum with subsequent population expansion in the Holocene and the future. Although genetic variation did not differ substantially across disturbed and undisturbed sites, there were small trends indicating higher genetic diversity and population bottlenecks in disturbed forests. As a result, disrupted ecosystems might display surprising genetic patterns that are difficult to predict and should not be overlooked.

## 1 Introduction

*Ailanthus altissima* Mill. Swingle, commonly known as the tree of heaven, belongs to the family Simaroubaceae, and is a deciduous tree species native to Eastern Asia (Kowarik and Säumel, 2007). This fast-growing tree species can reach a height of 18 to 21 m in just 10 years, has a life span of up to 130 years, and reproduces through both seeds and sprouts (Kowarik and Säumel, 2007). A single female tree can produce around a million seeds per year, with a high viability rate of 0.79 beyond approximately 5 years (Wickert et al., 2017). Although it is often found growing near forest edges, *A*. *altissima* is also commonly found inside forests (Rebbeck et al., 2017). *A*. *altissima* is highly valued for its medicinal, pharmaceutical, honey and paper production, essential oil source, erosion control, reforestation, land reclamation, ornamental, soil formation, and nutrient cycling purposes. Its low environmental requirements and rapid growth make it an ideal species for reclaiming degraded land or for environmental restoration projects (Sladonja et al., 2015). Because of these useful traits, *A*. *altissima* has been introduced to other continents for economic and ornamental purposes, except for Antarctica (Kowarik and Säumel, 2007; Sladonja et al., 2015). In Europe and North America, this plant has become an invasive species due to its rapid growth and high tolerance (Burch and Zedaker, 2003).

Anthropogenic activities such as hybridization, forest clearance, tree logging, planting improved trees, and climate change have posed numerous threats to forest ecosystems in tropical and temperate climates (Lamb et al., 2005; Bacles and Jump, 2011; Li et al., 2019; Chen et al., 2021). Genetic diversity of populations is crucial in determining their adaptability potential to future stressful environments and reflects past and present evolutionary bottlenecks (Hewitt, 2000; Reed and Frankham, 2003; Frankham, 2005; Yang et al., 2022). Human disturbances can either increase or maintain genetic variation, for instance, by increasing rates of mutation and connectivity between populations, providing environments favoring heterozygote advantage and hybridization, or magnifying temporal variation (DiBattista, 2008). However, human disturbances can also indirectly lower genetic diversity due to inbreeding and population isolation, and directly due to reduced population sizes and high mortality rates (Banks et al., 2013). The founder effect, changes in genetic structure and diversity, and degree of differentiation may occur in newly introduced populations, specifically led by purposeful or unintentional species translocation. Moreover, populations exhibit rapid life history evolution despite genetic bottlenecks (Dlugosch and Parker, 2008).

Population genetic studies of *A*. *altissima* have been extensively conducted outside its native range. These studies have utilized various molecular markers such as isozymes (Feret and Bryant, 1974), chloroplast DNA sequences (Kurokochi et al., 2012), and nuclear microsatellite markers (Aldrich et al., 2010; Kurokochi et al., 2014; Chuman et al., 2015; Brusa and Holzapfel, 2018; Neophytou et al., 2018). However, there is still a lack of understanding regarding the genetic variability of populations of this species in its native range. Only two studies have been conducted in the native region, one focusing on phylogeography and the other on microsatellite marker discovery (Liao et al., 2014; Saina et al., 2021). The phylogeography study by Liao et al. (2014) based on chloroplast DNA spacer regions found a wide distribution of unique and common haplotypes in mainland China, suggesting the need for more molecular methods to confirm the evolutionary history of *A*. *altissima*. Therefore, to fully comprehend the effects of human disturbance on *A*. *altissima* in its native range, population genetic studies using other molecular methods are necessary.

Simple sequence repeat (SSR) markers are highly polymorphic and widely distributed in eukaryotic genomes. Chloroplast microsatellites (cpSSRs) provide useful information about ancient historical relationships among maternal lines, while nuclear microsatellites are preferred in the evaluation of patterns of genetic structure due to their codominant and hypervariable nature. The present investigation aimed to estimate genetic variation and test for evidence of past bottlenecks on disturbed and undisturbed populations of *A*. *altissima* using 10 nuclear expressed sequence tag (EST)-derived microsatellite markers (EST-SSR) and 10 cpSSR markers. The study aimed to determine if disturbance would reduce, maintain, or increase the genetic diversity of *A*. *altissima* across undisturbed and disturbed populations in China. Additionally, niche modeling was used to explore probable shifts in habitat suitability of *A*. *altissima* within and beyond the present distribution to provide a framework for establishing new populations. This study helps to unravel the population genetic diversity and structure of *A*. *altissima* in a human-impacted forest system and provides necessary information for its management and use.

## 2 Materials and methods

### 2.1 Species sampling, DNA extraction, PCR amplification, and sequencing

Our study sampled 432 trees from 34 populations (defined by the geographic distribution) across most of the native range in China, with each population consisting of 4–20 individuals spaced at least 50 m apart (**Table 1**). We collected 130 individuals from undisturbed old growth stands of *A*. *altissima*, mostly growing in or near natural reserves of China, and 302 wild trees from disturbed habitats, such as semi-natural forests close to roads, villages, or towns, that have experienced varying levels of disturbance over the past 60 years (**Table 1**). Twenty-one populations were located in disturbed habitats. Following the modified CTAB method described by Doyle (1991), we extracted genomic DNA from *A. altissima* leaf samples dried on silica gel. We genotyped individuals using 10 cpSSR and 10 EST-SSR markers, as described by Saina et al. (2021). Each forward primer was fluorescently labeled with 6-FAM. To perform PCR, we diluted the isolated DNA 1:10 in ddH_2_O and stored it at −20°C. PCRs were conducted in a 20-µL volume containing 50–100 ng (1 µL) of template DNA, 0.5 µL of each primer, 0.2 µL of Taq polymerase enzyme, 0.5 µL of dNTPs, 2.5 µL of Taq buffer, and 11.3 µL of ddH_2_O. PCR amplification was performed as follows: an initial denaturation phase at 95°C for 5 min, followed by 35 cycles of denaturation for 30 s at 95°C, annealing for 40 s at 52–54°C, and extension at 72°C for 40 s, with a final extension step at 72°C for 10 min. We determined the primers that produced a single brilliant band using 2% agarose gel electrophoresis. The forward sequence of the primers that produced clear bands were chosen, their 5’ ends were tagged with 6-FAM fluorescent dye, and they were then employed for genotyping. We examined the microsatellite marker profiles of all *A*. *altissima* individuals using GeneMapper 4.0 (Applied Biosystems). We sized PCR amplicons using GeneScan 500 ROX (Applied Biosystems) on an ABI 3730 XL capillary electrophoresis sequencer, and calculated allele lengths using GeneMapper v.4.0 (Applied Biosystems, Foster City, California, USA).

**Table 1:** Locations, geographic coordinates, and habitat descriptions of the 34 studied populations of *A. altissima* in China.

Locations	Pop ID.	Sample size	Longitude	Latitude	Disturbance	Description of the sampling sites
Yichangtucheng, Hubei	YT	8	111.58	30.80	No disturbance	Roadside of the forest
Zhijiang, Hubei	ZJ	4	111.58	30.46	No disturbance	In the forest
Dalaoling, Hubei	DLL	5	110.92	31.06	No disturbance	In the nature reserve
Changyang, Hubei	CY	9	110.54	55.27	No disturbance	In the nature reserve
Dangyang, Hubei	DY	4	110.33	30.93	No disturbance	Roadside of the forest
Nanping, Fujian	NP	7	118.17	26.65	No disturbance	In the forest near the forestry school
Wuyishan, Fujian	WYS	12	115.93	35.91	No disturbance	In the nature reserve
Linan, Zhejiang	LA	20	119.67	30.23	No disturbance	In the forest
Jande, Zhejiang	JD	11	119.13	29.45	No disturbance	In the forest park
Gaozhai, Guangxi	GZ	16	110.66	25.47	No disturbance	In the nature reserve
Huaping, Guangxi	HP	7	109.92	25.62	No disturbance	In the nature reserve
Maerkang, Sichuan	MEK	16	102.19	31.91	No disturbance	In the forest
Xiangchen, Sichuan	XC	11	99.88	29.02	No disturbance	In the forest
Tai’an, Shandong	TS	18	117.10	36.21	Moderate interference	Roadside of the mountain scenic area
Cixian, Hebei	CX	10	114.37	36.36	Serious interference	Roadside of the county
Handan, Hebei	HD	11	114.47	36.61	Serious interference	Roadside of the railway station
Jingbian, Shaanxi	JB	8	112.47	37.88	Serious interference	Roadside of the national highway
Lanxian, Shanxi	LX	7	111.67	38.28	Moderate interference	In the village
Suide, Shaanxi	SD	19	110.26	37.50	Moderate interference	In the village
Yuncheng, Shanxi	YC	13	111.03	34.97	Serious interference	Wasteland near the Salt Lake
Pingyao, Shanxi	PY	14	113.02	37.21	Moderate interference	In the village
Nanyang, Henan	TBY	12	113.29	32.41	Mild interference	In a scenic spot
Xixia, Henan	XX	10	111.76	33.64	Mild interference	The old sector ridge scenic area
Lingbao, Henan	LB	15	110.67	34.53	Serious interference	Roadside of the national highway
Lanhansuo, Gansu	LH	6	103.79	36.07	Serious interference	Roadside of the national highway
Xiangshan, Beijing	XS	7	116.18	39.99	Moderate interference	In the mountain park
Wugongshan, Jiangxi	PX	21	114.15	27.47	Mild interference	The village near the mountain
Shucheng, Anhui	SC	20	116.99	31.53	Serious interference	Roadside of the national highway
Chuzhou, Anhui	CZ	20	118.29	32.30	Mild interference	In a scenic spot
Zijinshan, Jiangsu	NJ	20	118.85	32.04	Moderate interference	In the park
Xixiashan, Jiangsu	XXS	20	118.96	32.15	Mild interference	In the mountain scenic area
Suzhou, Jiangsu	ZZY	20	120.63	31.33	Moderate interference	In the park
Shangfangshan, Jiangsu	SFS	12	120.58	31.25	Mild interference	In the forest park
Jingdezhen, Jiangxi	JDZ	19	117.29	29.32	Serious interference	Roadside of the national highway

### 2.2 Chloroplast and nuclear SSR diversity analysis

We used the GENEPOP 4.3 software (Raymond, 1995) to assess deviations from the Hardy–Weinberg equilibrium (HWE) and genotypic linkage disequilibrium (LD) among all pairs of loci, with Bonferroni corrections. To estimate null allele frequency and polymorphic information content, we used the Cervus 3.0 program (Kalinowski et al., 2007), while FSTAT (Goudet, 2003) was used to check global *F*_ST_ by region and the inbreeding coefficient (*F*_IS_). It is important to note that the estimate of genetic differentiation can be affected by null alleles (Chapuis and Estoup, 2007), so we also calculated per population genetic differentiation [*F*_ST (FREENA)_ values] using the FREENA software (Chapuis and Estoup, 2007). For nuclear EST-SSR loci, we calculated diversity indices, including expected and observed heterozygosities (*nH*_e_/*nH*_E_ and *nH*_o_/*nH*_O_), total number of alleles (*N*_t_), allelic richness (*N*_e_/*N*_E_), mean allelic number (*N*_a_/*N*_A_), Shannon’s Information Index (I), and number of private alleles (*A*_P_), across all loci and populations using GenAlEx v.6.5 (Peakall and Smouse, 2012).

For each individual, we defined a haplotype as a unique combination of different allele lengths in all 10 cpSSR loci. We used the Haplotype Analysis software version 1.05 (Eliades and Eliades, 2009) to assess the genetic diversity of *A*. *altissima* populations by computing the number of haplotypes (*A*), effective number of haplotypes (*cpN*_E_), haplotype diversity (*cpH*_E_), number of private haplotypes (*P*), haplotypic richness (*R*_h_), and mean within-population genetic distance between haplotypes (*D* ^2^_sh_). We calculated the number of alleles using FSTAT software (Goudet, 2003), and assessed the relationships between haplotypes using the Neighbor-Net algorithm in SplitsTree4 (Huson and Bryant, 2006). We constructed a neighbor-joining tree using MEGA (Tamura et al., 2011) and displayed it using FigTree version 1.4.2 (Rambaut, 2014).

### 2.3 Population structure

We utilized Arlequin v3.5 to conduct the analysis of molecular variance (AMOVA) (Excoffier and Lischer, 2010). To determine whether the populations of *A*. *altissima* are differentiated due to the effects of isolation by distance (IBD), we used genetic and geographic distances to perform the Mantel test, executed in GeneAlEx 6.5 (Peakall and Smouse, 2012), with 10,000 permutations. The genetic distance population pairwise was used to perform the principal coordinate analysis (PCoA) in GenAlEx v 6.5 (Peakall and Smouse, 2012), and to draw the neighbor-joining tree in MEGA_X_ v.10.0.5 (Tamura et al., 2011), which was visualized using the program FigTree version 1.4.2 (Rambaut, 2014). Two approaches were used to determine the number of population clusters (*K*). The first method was performed in the STRUCTURE software v 2.3.4 (Pritchard et al., 2000) among the 34 populations of *A*. *altissima* with the admixture model and the assumption of correlated allele frequencies using 10 EST-SSR loci. Since most of the populations deviated from being in Hardy–Weinberg equilibrium (Szczecińska et al., 2016), the non-admixture model (independent allele frequencies) was also used. The highest probable number of clusters (*K*) was evaluated using 10 independent runs per *K*, by running *K* values from 1 to 34. Burn-in period was set to 50,000 and Markov Chain Monte Carlo (MCMC) length to 500,000 for each run. We utilized a web-based STRUCTURE HARVESTER 0.6.94 (Earl and Vonholdt, 2012) to find the optimal *K* value (number of groups) as well as the maximum delta *K* value (Evanno et al., 2005). CLUMPP v.1.1.2 (Jakobsson and Rosenberg, 2007) and DISTRUCT v.1.1 (Rosenberg, 2004) were used to combine the clusters across all replications and to visualize the outputs, respectively. The second complement to STRUCTURE analysis was a discriminant analysis of the principal component (DAPC) R package (Jombart, 2008). We applied the neighbor-joining tree algorithm for tree building. We tested the presence of phylogeographic patterns by comparing the *R*_ST_ and *G*_ST_ values within cpSSR loci using the SPAGeDi program 1.3 (Hardy and Vekemans, 2002) with 10,000 permutations.

### 2.4 Population bottlenecks

We assessed the population history of *A*. *altissima* using the BOTTLENECK v.1.2.02 program (Piry et al., 1999) to test for recent reductions in effective population size due to human disturbances. We implemented the two-phased model (TPM), which is highly preferred for microsatellite loci (Piry et al., 1999), by combining 70% of the infinite alleles model (IAM) with 30% of the stepwise mutation model (SMM), and tested for a 5% significance using Wilcoxon’s sign-rank test. Normally, deviations in mode shift presented as normal L-shaped distributions indicate no demographic bottleneck in populations, while a shift in mode designates otherwise.

### 2.5 Ecological niche modeling

We used MaxEnt 3.4.0 (Phillips and Dudík, 2008) to generate long-span ecological niche models (ENMs) for *A. altissima* across time (LGM-Future). The study area was defined by geographical coordinates of 20.9°–43.6°N and 91.5°–126°E. We obtained *A*. *altissima* locality data from the Chinese Virtual Herbarium (CVH, http://www.cvh.ac.cn/) and field data collected by Liao et al. (2014). We performed spatial rarefaction on the 142 records using the “spThin” v. 0.1.0 package in R (Aiello-Lammens et al., 2015), retaining 132 points for subsequent analyses with a minimum distance of 10 km between points.

We downloaded 19 bioclimatic variables and elevation (altitude) data with a resolution of 2.5 arc-min from WorldClim (http://www.worldclim.org/) (Hijmans et al., 2005). We performed a Pearson correlation analysis on the bioclimatic variables (*r* < 0.85) to remove correlated variables. For the LGM, Mid-Hol, and Future periods, we used calibrated global climate model (GCM) data from the Community Climate System Model “CCSM4”. The future period was represented by the representative concentration pathway 8.5 (RCP8.5) to represent pessimistic scenarios (Meinshausen et al., 2011). All ENM models were run with 10,000 background points and allowed to converge at 0.00001 after 5,000 iterations, using 10-fold replicates with bootstrap validation. Final models were exported and analyzed in ArcGIS 10.5.

## 3 Results

### 3.1 Genetic diversity

All EST-SSR loci observed significant HWE deviations (*p* < 0.001) except loci Ail 29 (**Table 2**), this was likely as a result of heterozygote excess (negative *F*_IS_ values) observed in all populations. A total of 255 alleles were yielded using the 10 EST-SSRs, varying from 7 to 36 in locus Ail 22 to locus Ail 06, respectively (average = 19.615). The mean expected heterozygosity (*nH*_e_) 0.807 and mean observed heterozygosity (*nH*_o_) 1.000 were highest at locus Ail 09 and Ail 26, respectively. The PIC values varied from 0.632 (locus Ail 11) to 0.908 (locus Ail 09) with a mean of 0.771 (**Table 2**). Estimates of genetic diversity at the population level are outlined in **Table 3**. The genetic diversity level of the 34 A*. altissima* populations was relatively high. The expected heterozygosity (*nH*_E_) and observed heterozygosity (*nH*_O_) values were moderate to high ranging from 0.547 to 0.772, and from 0.725 to 0.930, with an average value of 0.680 and 0.848, respectively. A total of 62 private alleles were realized in 34 populations, all with varying frequencies. Population XXS recorded the highest number of private alleles. For each population, the values of *N*_A_, *N*_E_, and inbreeding coefficient (*F*_IS_) ranged from 2.700 to 9.100, 2.344 to 5.330, and −0.459 to −0.097, respectively. Moreover, we found no significant difference in *F*_ST_ values (**Table S1**) when genotypes were corrected for null alleles by FREENA (Chapuis and Estoup, 2007).

**Table 2:** Genetic diversity of 10 EST-SSR loci within 34 populations of *A. altissima*.

Locus	*N*_t_	*N*_a_	*N*_e_	*nH*_o_	*nH*_e_	PIC	HWE	Accession No.
Ail 04-1	22	5.765	3.866	0.944	0.720	0.816	***	MN531157
Ail 06-3	36	7.000	4.430	0.777	0.734	0.892	***	MN531159
Ail 08-4	14	3.912	2.439	0.592	0.531	0.712	***	MN531160
Ail 09-5	33	8.618	5.770	0.948	0.807	0.908	***	MN531161
Ail 11-6	18	3.941	2.566	0.981	0.601	0.632	***	MN531162
Ail 19-7	29	8.059	5.268	0.863	0.782	0.902	***	MN531163
Ail 20-8	28	6.294	3.653	0.789	0.668	0.818	***	MN531164
Ail 25-10	14	4.412	2.943	0.779	0.637	0.81	***	MN531166
Ail 26-11	8	3.294	2.625	0.999	0.591	0.755	***	MN531167
Ail 29-13	27	6.676	4.122	0.804	0.722	0.884	NS	MN531169
**Average**	19.615	5.115	3.431	0.825	0.648	0.771		

*N_t_*, allelic number; *N_a_*, average number of alleles; *N_e_*, allelic richness; *nH_e_*, expected heterozygosity; *nH_o_*, observed heterozygosity; PIC, polymorphic information content; HWE, Hardy–Weinberg equilibrium deviations. Significant levels (***p < 0.001), NS, no significance.

**Table 3:** Genetic diversity estimates for 10 EST-SSRs in the investigated *A. altissima* populations.

Pop	*N*_A_	*N*_E_	*A*_p_	*I*	*nH*_O_	*nH*_E_	*F*_IS_	TPM	Mode shift
YT	4.500	3.067	2.000	1.208	0.788	0.636	−0.142	0.244	Normal
ZJ	2.700	2.344	0.000	0.885	0.850	0.556	−0.459	0.000***	Shifted
DLL	3.400	2.633	0.000	1.035	0.920	0.594	−0.392	0.002**	Shifted
CY	5.600	3.906	0.000	1.421	0.844	0.702	−0.138	0.127	Normal
DY	3.200	2.852	2.000	0.963	0.725	0.547	−0.306	0.000***	Shifted
NP	3.700	2.636	0.000	1.030	0.800	0.568	−0.352	0.151	Normal
WYS	5.500	3.620	0.000	1.404	0.883	0.697	−0.171	0.033*	Normal
LA	7.600	4.532	3.000	1.571	0.880	0.714	−0.144	0.376	Normal
JD	5.700	3.598	0.000	1.399	0.836	0.688	−0.170	0.414	Normal
GZ	7.100	4.263	3.000	1.500	0.894	0.702	−0.211	0.340	Normal
HP	5.200	3.672	2.000	1.365	0.900	0.683	−0.257	0.542	Normal
MEK	5.100	3.202	3.000	1.276	0.913	0.669	−0.351	0.080	Normal
XC	4.100	2.828	1.000	1.130	0.909	0.619	−0.417	0.216	Normal
TS	7.300	4.176	0.000	1.542	0.867	0.712	−0.153	0.340	Normal
CX	6.500	4.708	0.000	1.615	0.900	0.759	−0.146	0.003**	Normal
HD	6.600	4.287	1.000	1.582	0.864	0749	−0.103	0.021*	Normal
JB	5.800	3.901	2.000	1.466	0.825	0.706	−0.123	0.497	Normal
LX	4.800	3.622	2.000	1.314	0.871	0.670	−0.141	0.021*	Normal
SD	6.900	3.999	1.000	1.522	0.779	0.716	−0.097	0.735	Normal
YC	4.600	2.729	0.000	1.147	0.731	0.611	−0.229	0.244	Normal
PY	5.200	3.594	1.000	1.328	0.814	0.673	−0.185	0.003**	Normal
TBY	6.200	4.344	2.000	1.531	0.858	0.730	−0.198	0.057	Normal
XX	6.000	4.342	4.000	1.441	0.800	0.698	−0.131	0.021*	Normal
LB	6.700	3895	2.000	1.484	0.860	0.710	−0.233	0.542	Normal
LH	3.900	2.948	0.000	1.134	0.800	0.625	−0.191	0.033*	Normal
XS	4.700	3.452	2.000	1.258	0.800	0.643	−0.153	0.110	Normal
PX	8.600	4.798	7.000	1.739	0.857	0.772	−0.104	0.735	Normal
SC	9.000	5.330	5.000	1.767	0.905	0.768	−0.151	0.068	Normal
CZ	7.900	4.435	2.000	1.629	0.880	0.746	−0.142	0.273	Normal
NJ	7.200	4.540	2.000	1.576	0.865	0.720	−0.185	0.005**	Normal
XXS	9.100	5.297	8.000	1.731	0.855	0.739	−0.139	0.787	Normal
ZZY	4.800	3.191	1.000	1.260	0.930	0.663	−0.340	0.001***	Shifted
SFS	4.800	2.800	1.000	1.177	0.725	0.608	−0.164	1.000	Normal
JDZ	7.100	4.576	3.000	1.596	0.889	0.741	−0.163	0.021*	Normal
Mean	5.797	3.768	2.583	1.383	0.848	0.680	−0.205		

*A_P_*, number of private alleles; *N_A_*, number of alleles; *N_E_*, allelic richness; *I*, Shannon’s information index; *nH_E_*, expected heterozygosity; *nH_O_*, observed heterozygosity; *F_IS_*, inbreeding coefficient; Wilcoxon signed-rank tests for heterozygosity excess probabilities; TPM, two-phase model. Significant levels (*p < 0.05; **p < 0.01; ***p < 0.001).

Across the 10 cpSSR loci, a total of 92 different chloroplast alleles were recorded among the 34 studied populations of *A. altissima* (**Table 4**). The number of alleles for each locus varied between 4 at cpSSR12 and 18 at cpSSR2 and cpSSR9, with a mean of 9.2 alleles per locus. A combination of different size variants resulted in 292 different chloroplast haplotypes among 432 individuals of *A. altissima* from 34 studied populations. Approximately 90.75% (265/292) of the recorded haplotypes were private haplotypes. The most abundant haplotype was H1, which was found in 16 individuals, followed by H2 with 9 individuals; H3 to H64 were identified in 2 to 8 individuals, while the remaining haplotypes (H65 to H292) had single individuals each with a complex network (**Table S2**).

**Table 4:** Characteristics of the 10 cpSSRs evaluated in 34 A*. altissima* populations.

Locus	Total number of alleles	*R*_ST_	*G*_ST_	Size range (bp)	Accession no.
cpSSR1	5	0.848	0.495	267–284	MN531147
cpSSR2	18	0.265	0.578	222–276	MN531148
cpSSR5	6	0.592	0.464	256–263	MN531149
cpSSR6	5	0.472	0.421	250–276	MN531150
cpSSR7	10	0.570	0.554	218–279	MN531151
cpSSR8	14	0.665	0.387	277–299	MN531152
cpSSR9	18	0.366	0.623	254–306	MN531153
cpSSR10	5	0.712	0.603	266–275	MN531154
cpSSR12	4	0.526	0.444	108–111	MN531155
cpSSR13	7	0.275	0.345	247–65	MN531156
Mean	9.2	0.434	0.493		

*G_ST_* and *R_ST_* = population differentiation based on allele size.

Within *A. altissima* populations, PX had the highest number of haplotypes (*A* = 20), followed by LA and CZ populations with 18 haplotypes each, while three populations (ZJ, DY, and DLL) had the lowest number of detected haplotypes (*A* = 4). We detected a number of private haplotypes in 34 populations with a maximum of 20 in PX, followed by LA and CZ populations with 18 haplotypes each, and NJ and XXS with 15 and 14 haplotypes, respectively. Population MEK lacked the private haplotypes, while a minimum number of one haplotype was recorded in the XC population, and two for HD. The effective number of haplotypes (*cpN*_E_) was lowest in population SFS (3.273) and DLL (3.571), and highest in PX (*cpN*_E_ = 19.174), followed by populations LA and CZ (*cpN*_E_ = 16.667). Populations (ZJ, CY, DY, HP, LX, XX, and LH) had the highest haplotype richness (*R*_h_ = 3.000), whereas MEK, and SFS populations had the lowest (*R*_h_ = 1.859 and *R*_h_ = 1.879), respectively. The highest genetic diversity values were observed in populations ZJ, CY, DY, HP, LX, XX, and LH (*cpH*_E_ = 1.000), and the lowest in population SFS (*cpH*_E_ = 0.758). The average genetic distance between individuals ranged greatly from *D* ^2^_sh_ = 343.435 (population WYS) to *D* ^2^_sh_ = 1.800 (LX) (**Table 5**).

**Table 5:** Genetic parameters of the studied *A. altissima* populations based on 10 cpSSRs.

Pop	*A*	*P*	*cpN*_E_	*R*_h_	*cpH*_E_	*F*_ST_	*D* ^2^_sh_	TPM
YT	6	6	5.333	2.571	0.929	0.169	1.443	0.055*
ZJ	4	4	4.000	3.000	1.000	0.206	51.333	0.070*
DLL	4	4	3.571	2.400	0.900	0.224	0.620	0.668*
CY	9	9	9.000	3.000	1.000	0.127	1.850	0.308*
DY	4	4	4.000	3.000	1.000	0.206	79.667	0.022*
NP	6	6	5.444	2.714	0.952	0.166	77.467	0.141*
WYS	11	11	10.286	2.909	0.985	0.119	343.435	0.028
LA	18	18	16.667	2.937	0.989	0.100	26.072	0.505 (S)
JD	8	8	6.368	2.588	0.927	0.151	16.953	0.276 (S)
GZ	11	10	9.143	2.707	0.950	0.125	74.389	0.294
HP	7	7	7.000	3.000	1.000	0.144	4.610	0.112*
MEK	6	0	3.556	1.859	0.767	0.213	0.269	0.312 (S)
XC	6	1	3.667	2.027	0.800	0.207	0.269	0.665
TS	11	8	6.231	2.426	0.889	0.151	91.618	0.070 (S)
CX	8	5	6.250	2.633	0.933	0.152	134.002	0.432 (S)
HD	9	2	7.118	2.697	0.945	0.132	221.858	0.188
JB	7	4	6.400	2.786	0.964	0.147	15.525	0.433*
LX	7	4	7.000	3.000	1.000	0.142	1.800	0.570*
SD	16	11	13.370	2.864	0.977	0.106	4.119	0.549
YC	11	4	8.895	2.783	0.962	0.123	19.838	0.121 (S)
PY	11	6	8.909	2.747	0.956	0.125	0.809	0.604
TBY	11	10	10.286	2.909	0.985	0.119	22.758	0.582
XX	10	5	10.000	3.000	1.000	0.119	1.849	0.237 (S)
LB	10	5	6.818	2.529	0.914	0.142	76.998	0.104
LH	6	6	6.000	3.000	1.000	0.157	39.047	0.568*
XS	6	4	5.444	2.714	0.952	0.165	158.886	0.180*
PX	20	20	19.174	2.971	0.995	0.096	78.292	0.507
SC	17	11	14.286	2.877	0.979	0.104	111.507	0.405
CZ	18	18	16.667	2.937	0.989	0.100	95.663	0.228
NJ	16	15	13.333	2.846	0.974	0.108	283.130	0.279 (S)
XXS	14	14	11.765	2.782	0.963	0.113	4.644	0.500
ZZY	10	10	4.167	2.069	0.800	0.199	6.394	0.537 (S)
SFS	6	5	3.273	1.879	0.758	0.240	8.327	0.675
JDZ	9	9	5.730	2.301	0.871	0.161	17.137	0.552
Mean	9.794	7.765	8.210	2.690	0.941	0.147	60.958	

*A*, number of haplotypes; *P*, number of private haplotypes; *cpN_E,_* effective number of haplotypes; *R_h_*, haplotypic richness; *cpH_E,_* genetic diversity; *D^2^_sh_*, mean genetic distance between samples, *p < 0.05.

### 3.2 Population structure

**Figure 1:**
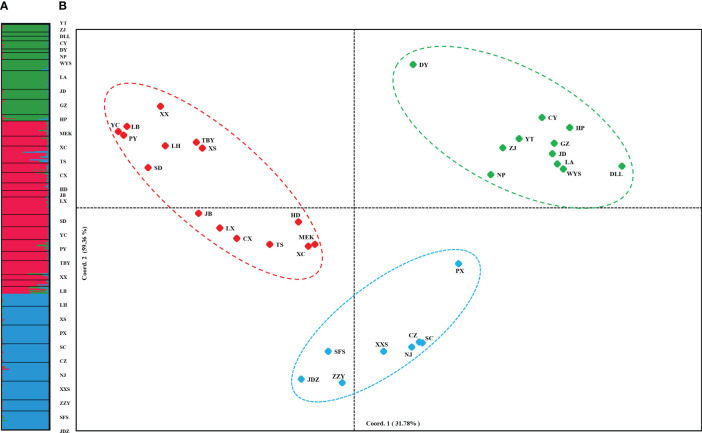
**(A)** STRUCTURE clustering results for 432 *A. altissima* individuals from 34 populations based on variation at 10 EST-SSR markers. Histogram of individual assignments and different populations are separated by black lines, each color corresponds to a suggested cluster, and population codes are indicated below. **(B)** PCoA based on pairwise Nei’s standard genetic distances sorted by populations.

**Figure 2:**
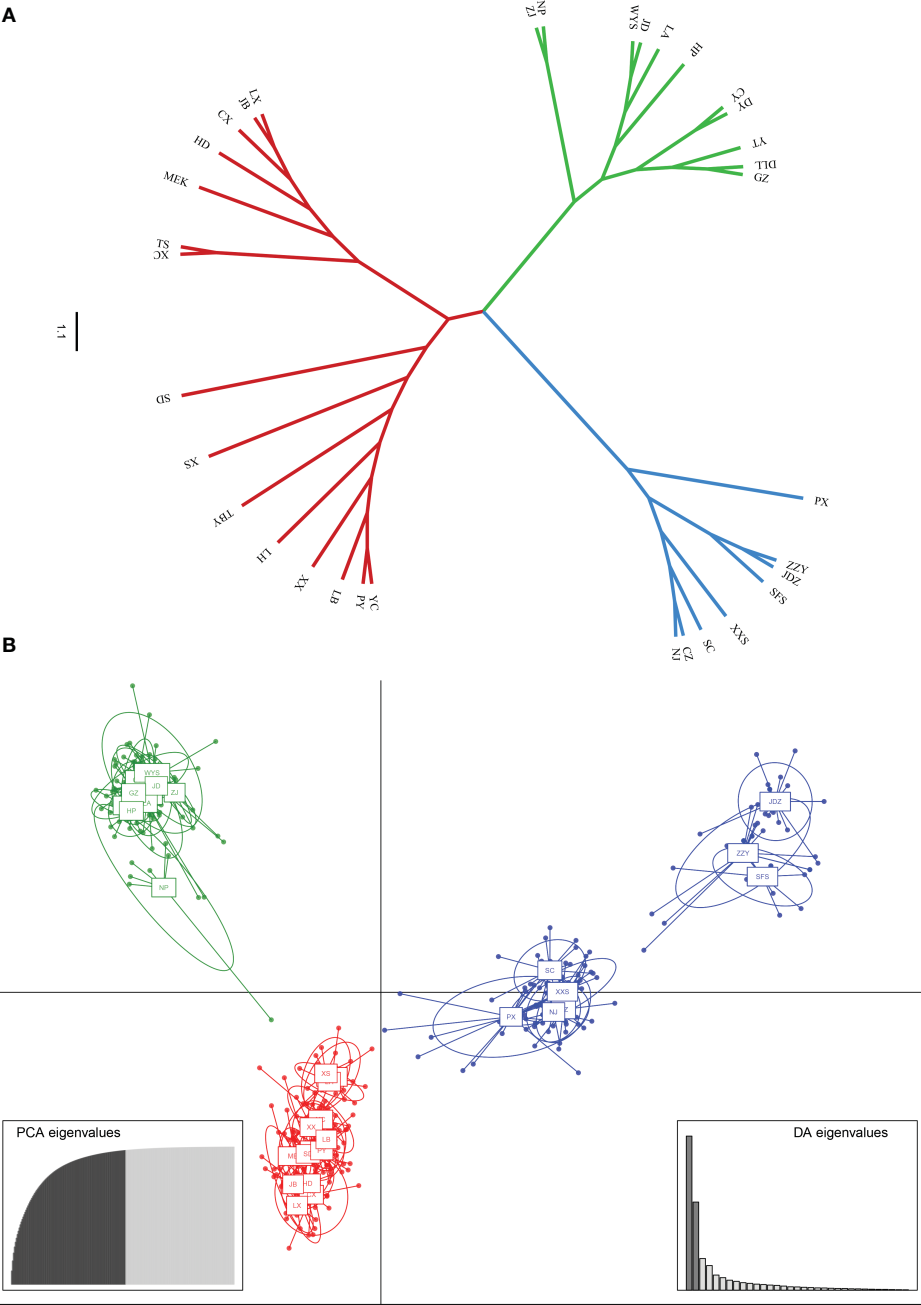
**(A)** Neighbor-Joining tree showing relationships of 34 studied populations of *A. altissima.* **(B)** Scatter plot of discriminant analysis of principal components using 10 EST-SSRs of *A*. *altissima*.

**Figure 3:**
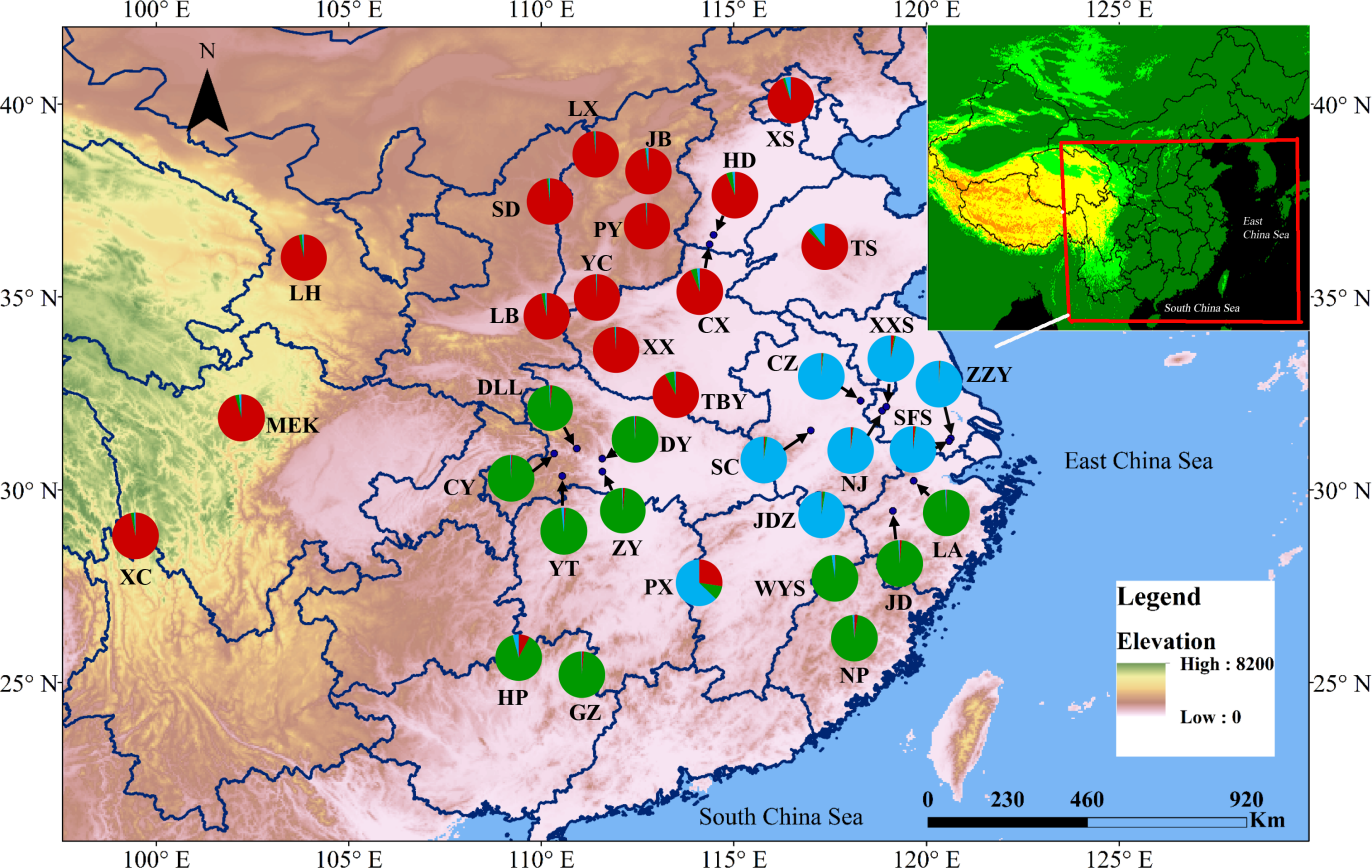
Map showing the sampling locations of the 34 populations of *A. altissima* at 10 EST-SSRs loci and their color-coded structuring when *K*= 3.

**Figure 4:**
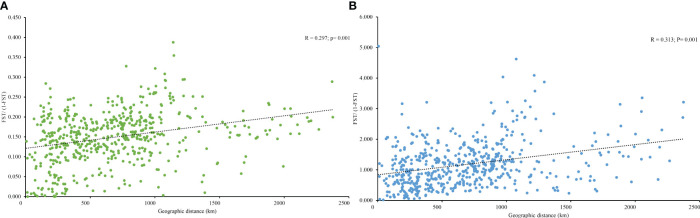
**(A)** Relationship between the pairwise *F*_ST_/(1 − *F*_ST_) (isolation by distance analysis) and geographic distance in *A. altissima* based on 10 EST-SSR markers and **(B)** 10 cpSSR markers.

PCoA results were consistent with STRUCTURE results (**Figures 1A, B**). The first and the second axis accounted for 25.81% and 20.40%, respectively (**Figure 1B**). Using Nei’s genetic distance matrix, the 34 populations were divided into three main branches using the neighbor-joining method (**Figure 2A**). STRUCTURE (**Figure 1A** and **Figure S1**), PCoA (**Figure 1B**), neighbor-joining tree (**Figure 2A**), and DAPC (**Figure 2B**) analyses yielded similar results, indicating consistency and reliability in the results. The 34 populations were clustered into three main groups from the 10 EST-SSRs (**Figures 1**–**3**). The first included 15 populations (TBY, LB, PY, YC, XX, SD, LX, LH, MEK, XC, JB, CX, HD, TS, and CX), the second group comprised 8 populations (NJ, CZ, SC, XXS, SFS, JDZ, ZZY, and PX), and the third group consisted of 11 populations (ZJ, NP, WYS, JD, LA, HP, CY, DY, YT, DLL, and GZ). The neighbor-joining (NJ) tree based on Nei’s genetic distance clearly identified five main clusters at 10 cpSSR loci (**Figure S2**). The first cluster consisted of 16 populations (CY, JD, ZJ, WYS, PX, DY, NJ, JDZ, PY, XX, TBY, YC, LB, GZ, CX and SC), the second contained 10 populations (NP, XXS, LX, SD, TS, XS, JB, HD, MEK, and XC), and the third included 6 populations (YT, DLL, CZ, ZZY, HP, and LH), while the fourth and fifth had a single population each, LA, and SFS, respectively. Based on 10 EST-SSR markers, AMOVA results revealed that a higher genetic variation was mostly resided within populations at the disturbance level (85.62%) and overall (87.40%), respectively. Mantel test results revealed a significant correlation between Slatkin’s linearized *F*_ST_ and geographic distance *(r* = 0.309, *p* = 0.002) (**Figure 4**). The global *nF*_ST_ value was 0.126, while a 0.144 differentiation represented the disturbance level (**Table 6**).

**Table 6:** Hierarchical analyses of molecular variance (AMOVA) of *A. altissima* populations based on nuclear and chloroplast microsatellites.

EST-SSRs						
Group	Source of variation	d.f.	S.S	V.C	P.V	
Global	Among populations	33	742.164	0.69823 Va	12.60	*nF*_ST_ = 0.126***
	Within populations	830	4,020.327	4.84377 Vb	87.40	
Disturbance	Among groups	1	95.554	0.20443 Va	3.61	*nF*_ST_ = 0.144***
	Among populations	32	646.61	0.69823 Vb	10.77	
	Within populations	830	4,020.327	4.84377 Vc	85.62	
	Total	863	4,762.491			
cpSSRs						
Global	Among populations	33	1,369.525	1.58170 Va	50.87	*cpF*_ST_ = 0.509***
	Within populations	830	1,267.776	4.84377 Vb	49.13	
Disturbance	Among groups	1	56.846	0.03621 Va	1.16	*cpF*_ST_ = 0.512***
	Among populations	32	1,312.679	1.56591 Vb	50.04	
	Within populations	830	1,267.776	1.52744 Vc	48.81	
	Total	863	2,637.301			

Significance level (***p = 0.001); d.f., degree of freedom; S.S., sum of squares; V.C., variance components; P.V., percentage variation.

### 3.3 Recent bottlenecks

Mutation-drift equilibrium tests revealed that 13 populations (WYS, CX, HD, PY, XX, NJ, ZZY, JDZ, ZJ, DLL, DY, LX, and LH) significantly deviated from the three-phase model (TPM) equilibrium (**Table 2**). Among these populations, disturbances were observed in most of them (HD, PY, XX, NJ, ZZY, JDZ, LX, and LH) (**Table 3**). However, only populations ZJ, DLL, DY, and ZZY exhibited distortions of the mode shift (shifted mode) (**Table 3**). For the 10 cpSSRs, populations LA, JD, MEK, CX, TS, YC, XX, NJ, and ZZY displayed shifted mode, with six populations (CX, TS, YC, XX, NJ, and ZZY) from disturbed regions (**Table 5**).

### 3.4 Ecological niche modeling

**Figure 5:**
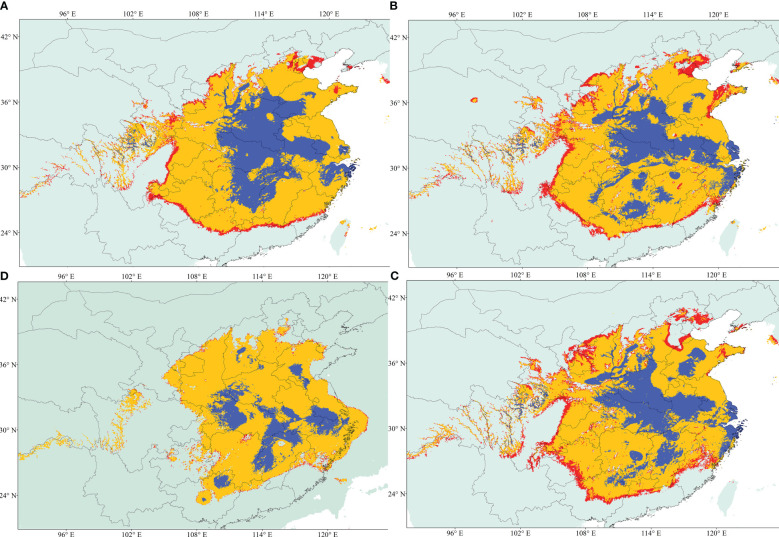
Geographical distribution models showing climatic suitability for *A. altissima*. **(A)** Future; **(B)** Current; **(C)** Mid-Holocene; and **(D)** Last Glacial Maximum (LGM). Habitat suitability is shown according to the colors (blue, logistic value 0.6 to 1; red, logistic value 0.25).

Eight of the 19 variables were selected for constructing the ecological niche models (ENMs) for *A*. *altissima* after conducting a Pearson correlation analysis (**Table S3**). The performance of our models was assessed using AUC-Maxent values. A model is considered poor if the AUC is less than 0.60, average if it falls between 0.60 and 0.70, and excellent if it exceeds 0.80. Based on this criterion, all of our habitat suitability models for *A*. *altissima* were deemed excellent (**Table S4**). **Figure 5** displays the ENMs predicting the distribution of habitat suitability for *A*. *altissima* in the present, past, and future. The habitat suitability distributions for *A*. *altissima* in the present period were found to be significantly higher than the known occurrences from our sampling collections and existing records. This outcome is not surprising, given the limited sampling conducted in Central China. Overall, *A*. *altissima* exhibited a wide range of habitat suitability across several provinces. The ENM models for the Last Glacial Maximum (LGM) period (22 kya; **Figure 5D**) predicted a notable eastward shift in the distribution of habitat suitability for the persistence of *A*. *altissima*, while also highlighting potential fragmented refugia in central and eastern China. This modeling demonstrated that many provinces are areas of high potential distribution for *A*. *altissima*, with the blue areas (**Figure 5**) generally being smaller and restricted to highland regions. Surprisingly, the ENM model predicts similar distributions of *A*. *altissima* during the Mid-Holocene (6 kya) compared to the LGM, indicating the presence of fragmented refugia during this period as well. Future projections for the year 2070 suggested a reduction in habitat suitability for *A*. *altissima* in the northern and southern regions, with a slight northeastern shift and a decrease in the number of suitable pixels.

## 4 Discussion

In general, wind-pollinated tree species exhibit high levels of genetic diversity within populations, reduced differentiation among populations, and exceptionally high outcrossing rates (Hamrick and Godt, 1996; Dow and Ashley, 1998). Consequently, pollen flow plays a crucial role in maintaining connectivity and genetic diversity among populations (Burczyk and Chybicki, 2004; Hamrick, 2004). However, human activities have significantly disturbed natural ecosystems for agricultural and urban purposes (Ottewell et al., 2009). Consequently, these habitat disturbances may result in the erosion of genetic diversity and an increase of population genetic divergence (Young et al., 1996; Liu et al., 2020). The consequences of habitat degradation include disruptions in the breeding systems and genetic bottlenecks of plants at both the individual and population levels (Jump and Peñuelas, 2006). Nevertheless, there is still much to be learned about genetic diversity in human-impacted populations of *A*. *altissima* within its native range. Compared to neutral nuclear SSRs, EST-SSRs were designed to amplify the microsatellite loci that are tightly associated with the transcribed regions of gene(s) (Varshney et al., 2005) and very useful for exploring the possible signatures of divergent selection in genome (Namroud et al., 2008). In our study, we found that almost all the EST-SSR markers were significantly deviated HWE (*p* < 0.001) except only one loci (Ail 29), suggesting that these EST-SSRs are mostly not neutral markers. Thus, these markers represent suitable molecular tools for assessing the impact of human disturbance (selection) on the genetic diversity of *A*. *altissima* across undisturbed and disturbed forests in China. Our study, utilizing nuclear EST-SSR markers, revealed moderate to high levels of genetic variation in both disturbed and undisturbed populations. The overall level of genetic diversity (*nH*_E_ = 0.680) found in this study is comparable to the *A. altissima* populations in Eastern United States (*H*_E_ = 0.629; Aldrich et al., 2010), and slightly lower than the Japanese populations (*H*_E_ = 0.740; Kurokochi et al., 2014) as well as populations investigated by Brusa and Holzapfel (2018; *H*_E_ = 0.939). The overall genetic diversity of *A*. *altissima* is also similar to that of several tree species found in subtropical China, with their distribution ranges partly overlapping with that of *A*. *altissima*, such as *Castanopsis fargesii* (*H*_E_ = 0.655; Li et al., 2014), *Phoebe bournei* (*H*_E_ = 0.651; Zhou et al., 2021), and *Camellia sinensis* (*H*_E_ = 0.640; Yao et al., 2012). However, it is lower than the genetic diversity of the relict plant *Ginkgo biloba* (*H*_E_ = 0.808; Zhou et al., 2020) and higher than that of the *Lindera glauca* (*H*_E_ = 0.520; Zhu et al., 2020), a dioecious species endemic to subtropical/warm-temperate forests of East Asia.

The overall genetic diversity (*cpH*_E_) of the 10 cpSSR loci was found to be 0.921. Our findings provide support for the idea that habitat disturbance in wind-pollinated trees promotes pollen movement, leading to increased genetic variation in the remaining populations (Bacles et al., 2005). In the case of *A*. *altissima*, the observed genetic variation can be attributed to the availability of suitable habitats resulting from human disturbance. This aligns with previous research suggesting that *A*. *altissima* thrives in areas with increased light availability, such as those created through timber harvesting, while dense forests with low-light conditions are less favorable for its survival (Call and Nilsen, 2003; Martin et al., 2010). Additionally, Brusa and Holzapfel (2018) proposed that areas with higher levels of human disturbance would exhibit greater genetic variation in introduced habitats, whereas areas with lower disturbance would show reduced diversity. Our study supports this hypothesis, as anthropogenic activities were found to influence the genetic diversity of many *A*. *altissima* populations.

When sample size is taken into account, estimations of private and allelic richness are comparable across the study populations. By concatenating the 10 cpSSRs, we detected a total of 292 haplotypes across 432 individuals of *A. altissima*. Diversity estimates showed lower values within each population (*H*_S_ = 0.847) than overall haplotypic variation (*H*_T_ = 0.993); this was supported by slightly higher differentiation values of *G*_ST_ = 0.493 compared to *R*_ST_ = 0.434, suggesting a weak phylogeographic structure. On the other hand, an increased number of microsatellites led to a higher haplotypic diversity than a previous study by Liao et al. (2014). High genetic diversity and number of haplotypes found within the *A. altissima* is comparable with other plant species studied with cpSSR markers, such as *Quercus semiserrata* (*H*_S_ = 0.16, *H*_T_ = 0.93) (Pakkad et al., 2008), three *Eucalyptus* species (*H*_S_ = 0.12–0.27, *H*_T_ = 0.64–0.92) (Nevill et al., 2014), and *H*_S_ = 0.74, *H*_T_ = 0.98 for *Tithonia rotundifolia* (López-Caamal et al., 2019). The life history of *A*. *altissima* may have contributed to the high number of detected haplotypes and their low frequency. This tree species is commonly found in disturbed habitats, and human-mediated seed dispersal may have played a role in its distribution. Additionally, *A*. *altissima* is a pioneer species that can withstand significant levels of human and natural disturbances, making it well-suited for disturbed sites and harsh weather conditions (Kowarik and Säumel, 2007; Constán-Nava et al., 2010). It is not surprising, therefore, that *A*. *altissima* seeds have a high potential for invading disturbed areas and can travel distances of over 200 m (Landenberger et al., 2007). Interestingly, Rebbeck and Jolliff (2018) found that *A*. *altissima* seeds can remain viable in forested ecosystems for more than 6 years under leaf litter, and human disturbances, such as timber harvesting, may have facilitated the spread of these seeds, subsequently improving conditions for seedling establishment. Overall, our results indicate that the genetic diversity of *A*. *altissima* was similar across populations, regardless of the level of disturbance. This suggests that, similar to other plants with both asexual and sexual reproduction systems, sexual events in *A*. *altissima* are sufficient to generate a consistent pattern of allelic variation and maintain genetic diversity in disturbed habitats, as observed in fully sexually reproducing individuals (Bengtsson, 2003; Latutrie et al., 2019).

Generally, outcrossing plant species have *nF*_ST_ values of 0.2 or less, with high genetic diversity within the populations, especially dioecious species (Nybom and Bartish, 2000; Cabrera-Toledo et al., 2008). In this study, a low genetic differentiation among populations was found in *A*. *altissima* with EST-SSRs (*nF*_ST_ = 0.126). As a dioecious species, *A*. *altissima* possess an outcrossing mating system and a long life cycle, which may have resulted in the low among-population and high within-population genetic variation. The current genetic differentiation results are similar to those reported by other studies that have used nuclear SSR markers, for instance, *Q. semiserrata* (*nF*_ST_ = 0.12) (Pakkad et al., 2008), *Q. castanea* (*nF*_ST_ = 0.13) (Valencia-Cuevas et al., 2014), *Prunus africana* (*nF*_ST_ = 0.12) (Mihretie et al., 2015), *Q. infectoria*, (*nF*_ST_ = 0.12) (Mohammad-Panah et al., 2017), and *Quercus* section *Lobatae* subsection *Racemiflorae* (*nF*_ST_ = 0.12) (McCauley et al., 2019).

In degraded ecosystems, genetic variation plays a crucial role in populations adapting to different environments (Huang et al., 2018). According to Mosca et al. (2012), the genetic diversity and structure of forest tree species are influenced by environmental, climatic, and geographical factors. In our study, we found a significant correlation between geographic distance and pairwise genetic differentiation (*F*_ST_) at a range-wide scale (**Figure 4**), indicating that natural dispersal has played a key role in shaping the pattern of IBD, unlike human-assisted dispersal. Another possible explanation for the observed IBD pattern is the colonization of different glacial refuges. Interestingly, populations from the northern and eastern regions formed distinct clusters, further supporting the presence of IBD. However, previous studies have reported no significant correlation between geographic distance and genetic diversity in introduced ranges (Kurokochi et al., 2014; Brusa and Holzapfel, 2018; Neophytou et al., 2019), as well as in cpDNA regions in China (Liao et al., 2014). The complex climatic and geographical features, along with the size of our study area, may have contributed to these different results (Huang et al., 2018). Similar inconsistent findings have been reported in other species, such as *Pinus tabulaeformis* (Wang and Hao, 2010), *Larix gmelinii* (Zhang et al., 2013), and *L. principis-rupprechtii* (Di et al., 2020).

Bottleneck analyses based on EST-SSRs indicate that the genetic diversity of some *A*. *altissima* populations has been impacted by a reduction in effective population size. Evidence of a recent bottleneck was observed in 13 out of 34 populations of *A*. *altissima* (**Table 3**). These findings are further supported by changes in mode shift using cpSSR markers in certain populations (**Table 5**), suggesting that *A*. *altissima* remnants have experienced a significant decrease in their effective population sizes in the recent past and are no longer in mutation-drift equilibrium. Consequently, a small effective population size may have led to an excess of heterozygotes in several populations. The absence of selfing in individuals results in an excess of heterozygotes, and a small number of breeders in a population can cause a slight difference in allelic frequencies between females and males due to binomial sampling error (Balloux, 2004). Previous research on microsatellites has also indicated that the genetic diversity of *A*. *altissima* may have been influenced by population bottlenecks in an area of early introduction (Neophytou et al., 2019). Therefore, the differences in genetic variation observed in this study may be shaped by various factors, such as human disturbances and climatic conditions.

Numerous studies have been conducted to investigate the effects of Qinghai–Tibet plateau uplifts on the climate in the warm temperate and subtropical zones of China. These studies have revealed that the plateau acted as a barrier against glaciation, resulting in arid climatic conditions during the quaternary period (Liang et al., 2019). The distribution model used in this study indicates that the range of *A*. *altissima* has expanded, while it contracted during the Mid-Holocene (182,914.765 km^2^) and the Last Glacial Maximum (766,327.534 km^2^). Therefore, this study supports the hypothesis that plants were influenced to some extent by the glaciation period and were able to persist during inter-glacial cycles. Furthermore, the findings suggest the presence of fragmented refugia during the Last Glacial Maximum and Mid-Holocene. This finding aligns with previous research indicating that the Qinghai–Tibet plateau acted as a protective barrier, allowing plants to survive the ice age by seeking refuge in central China (Liang et al., 2019; Ren et al., 2020). The species modeling results also confirm previously proposed refugial areas for *A*. *altissima* based on the phylogeographic study conducted by Liao et al. (2014).

## 5 Conclusions

To the best of our knowledge, no study has yet analyzed the population structure and genetic diversity of *A*. *altissima* in its native range using cpSSR and EST-SSR markers. Therefore, this study represents the first investigation into the genetic diversity of *A*. *altissima* in human-disturbed forests. Overall, we observed slight trends indicating reduced or increased levels of genetic diversity in disturbed areas. As a result, caution should be exercised when making generalizations about the response of plant genetic diversity in highly anthropogenically disturbed forests. The higher genetic diversity observed in disturbed areas may be attributed to seedling regeneration or the presence of individuals from multiple sources, which contributes to the maintenance of higher genetic diversity. A study by Rebbeck et al. (2017) supports our findings and enhances our understanding of *A*. *altissima’*s response to disturbances. Additionally, a comparative dendroecological study comparing *A*. *altissima* populations in native and invaded habitats provides valuable information that can help mitigate the rate of invasion or prevent the naturalization of this species in introduced ranges and disturbed areas (Knüsel et al., 2019). Furthermore, our study’s results are consistent with previous reports that indicate weak population structure based on cpSSRs, although we observed high genetic variation. Additionally, our modeling results provide evidence for population range contraction during the Last Glacial Maximum (89,705.518 km^2^), followed by population expansion in the Holocene (198,321.113 km^2^) and projected future (134,751.066 km^2^). In conclusion, the information regarding the extent of differentiation and genetic variation between and within *A*. *altissima* populations can aid in the future monitoring of this species in the face of ongoing environmental changes and human impacts.

## Data availability statement

The datasets presented in this study can be found in online repositories. The names of the repository/repositories and accession number(s) can be found below: https://www.ncbi.nlm.nih.gov/genbank/, MN531147 - MN531169.

## Author contributions

Y-YL and J-MC conceived and designed the experiment; JS performed the experiments, analyzed data, and visualized and wrote the first draft of the manuscript. Z-ZL and BN assisted in data analysis. RG, Y-YL, J-MC, and Z-ZL reviewed, edited, and commented on previous versions of the manuscript. All authors contributed to the article and approved the submitted version.
