# Supplementary material for: Exploring the genetic diversity and population structure of Ailanthus altissima using chloroplast and nuclear microsatellite DNA markers across its native range
Source: Front Plant Sci. 2023 Nov 22;14:1197137. doi: 10.3389/fpls.2023.1197137 (PMC10702488; doi:10.3389/fpls.2023.1197137)
Supplement: Supplementary file 1 [file DataSheet_1.docx]

Supplementary Material

Exploring the genetic diversity and population structure of *Ailanthus altissima* using chloroplast and nuclear microsatellite DNA markers across its native range.

Josphat K. Saina ^1, 2, 3, 4, 6^, Zhizhong Li ^2, 3^, Boniface K. Ngarega ^3, 4, 6^, Robert W. Gituru ^5^, Jinming Chen ^2, 3,^ * and Yiying Liao ^1,^ *

^1^ Fairy Lake Botanical Garden, Shenzhen & Chinese Academy of Sciences, Shenzhen 518004, China

^2^ Wuhan Botanical Garden, Chinese Academy of Sciences, Wuhan 430074, China

^3^ University of Chinese Academy of Sciences, Beijing 100049, China

^4^ Sino-African Joint Research Center, Chinese Academy of Sciences, Wuhan 430074, China

^5^ Department of Botany, Jomo Kenyatta University of Agriculture and Technology, Nairobi 62000-00200, Kenya

^6^ Center for Integrative Conservation, Xishuangbanna Tropical Botanical Garden, Chinese Academy of Sciences, Menglun, 666303 Yunnan, China

*** Correspondence:**Jinming Chen Email: ([jmchen@wbgcas.cn](mailto:jmchen@wbgcas.cn)), Yiying Liao ([liaoyiying@szbg.ac.cn](mailto:liaoyiying@szbg.ac.cn))

# Supplementary Figures and Tables

**Supplementary Tables**

**Table S3.** Bioclimatic variables used in modeling the distribution *Ailanthus altissima*

| Code | Variables | Unit | Source |
| --- | --- | --- | --- |
| Bio1 | Annual Mean Temperature | °C * 10 | WorldClim |
| **Bio2** | **Mean Diurnal Range (Mean of monthly (max temp - min temp))** | **°C * 10** | WorldClim |
| **Bio3** | **Isothermality** | **-** | WorldClim |
| Bio4 | Temperature Seasonality | - | WorldClim |
| Bio5 | Max Temperature of Warmest Month | °C/month | WorldClim |
| Bio6 | Min Temperature of Coldest Month | °C/month | WorldClim |
| Bio7 | Temperature Annual Range | °C | WorldClim |
| **Bio8** | **Mean Temperature of Wettest Quarter** | **°C/quarter** | WorldClim |
| **Bio9** | **Mean Temperature of Driest Quarter** | **°C/quarter** | WorldClim |
| Bio10 | Mean Temperature of Warmest Quarter | °C/quarter | WorldClim |
| Bio11 | Mean Temperature of Coldest Quarter | °C/quarter | WorldClim |
| Bio12 | Annual precipitation | mm/month | WorldClim |
| **Bio13** | **Precipitation of Wettest Month** | **mm/month** | WorldClim |
| **Bio14** | **Precipitation of Driest Month** | **mm/month** | WorldClim |
| **Bio15** | **Precipitation Seasonality (Coefficient of Variation)** | **-** | WorldClim |
| Bio16 | Precipitation of Wettest Quarter | mm/quarter | WorldClim |
| Bio17 | Precipitation of Driest Quarter | mm/quarter | WorldClim |
| **Bio18** | **Precipitation of Warmest Quarter** | **mm/quarter** | WorldClim |
| Bio19 | Precipitation of Coldest Quarter | mm/quarter | WorldClim |

**Table S4:** Predictive abilities of species distribution model (SDM) for *A. altissima*

| **Period** | **AUC-Maxent** | **MTSS** |
| --- | --- | --- |
| LGM | 0.964 | 0.290 |
| Mid-Hol | 0.952 | 0.229 |
| Current | 0.949 | 0.238 |
| Future (2070) | 0.952 | 0.256 |

**Supplementary Figures**


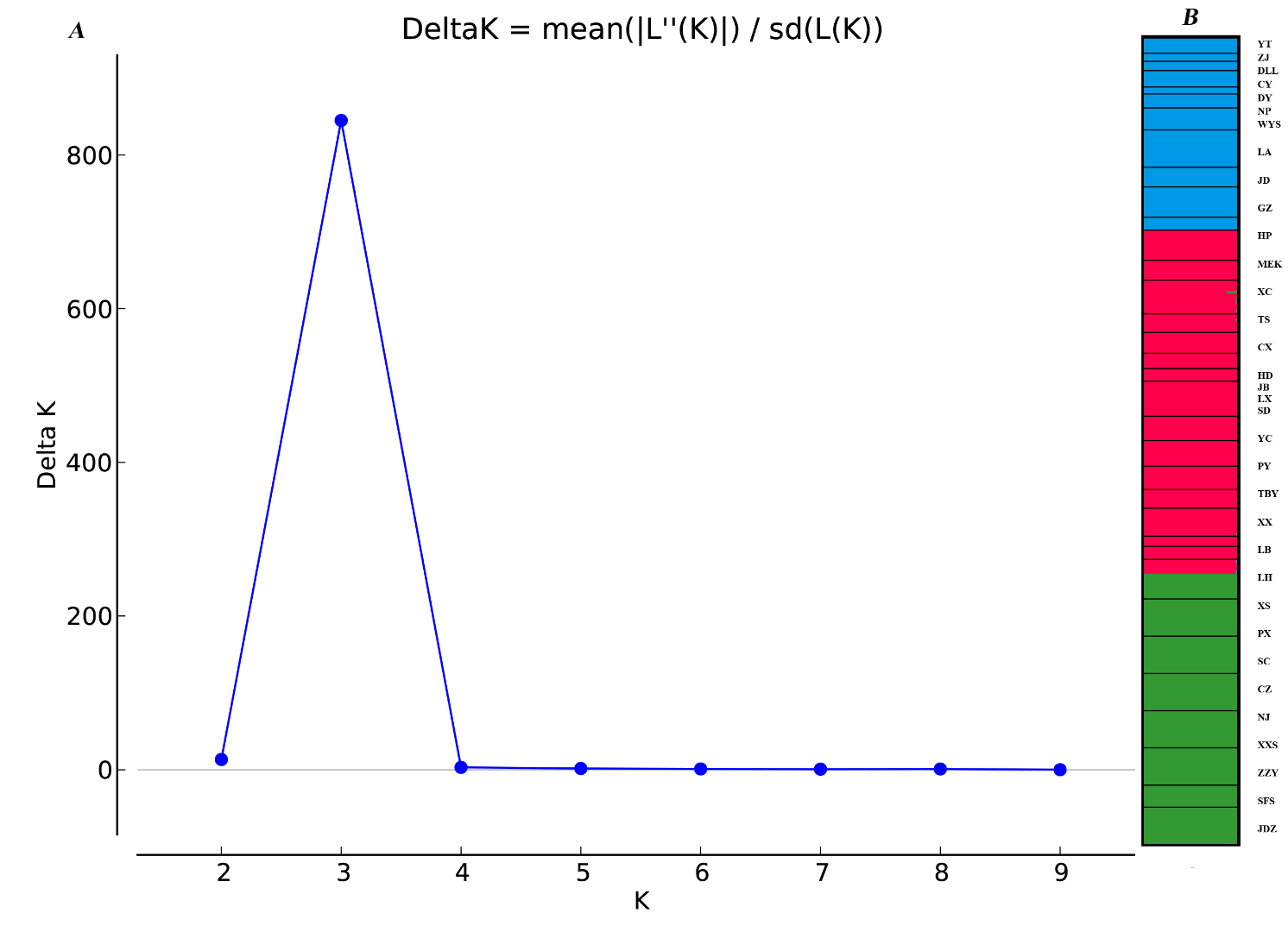
**Figure S1** A-The Optimal *K* value with the most suitable population clusters determined according to the values of *ΔK* estimated by Structure Harvester. B- non-admixture *K* = 3


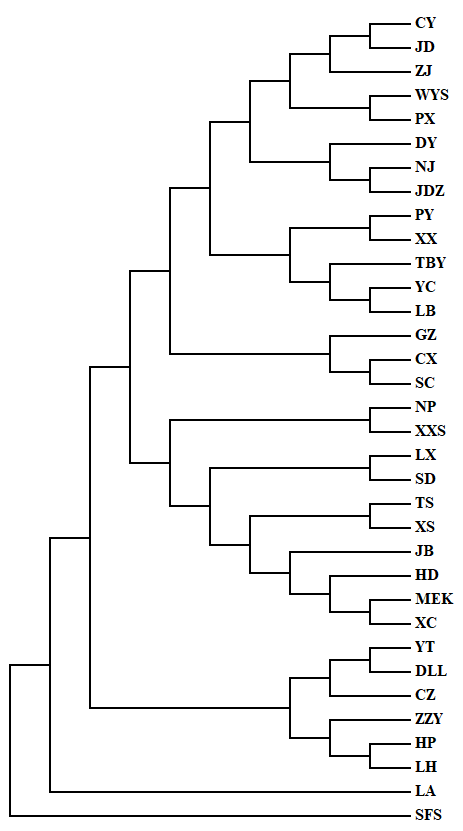


**Figure S2** Cluster dendrogram based on Nei’s genetic distance for cpSSR markers
